# Pulmonary nodule localization guided by computed tomography using a nitinol guidewire before video-assisted thoracoscopic surgery

**DOI:** 10.1590/0100-3984.2018.0040

**Published:** 2019

**Authors:** Tiago Kojun Tibana, Tony Rocha de Carvalho, Adalberto Arão Filho, Edson Marchiori, Thiago Franchi Nunes

**Affiliations:** 1 Hospital Universitário Maria Aparecida Pedrossian da Universidade Federal de Mato Grosso do Sul (HUMAP-UFMS), Campo Grande, MS, Brazil.; 2 MS Diagnósticos Médicos, Campo Grande, MS, Brazil.; 3 Universidade Federal do Rio de Janeiro (UFRJ), Rio de Janeiro, RJ, Brazil.

## INTRODUCTION

Image-guided puncture techniques are being used with increasing frequency in interventional radiology^(^^1^^-^^4^^)^. The appropriate management of a pulmonary nodule usually requires a definitive pathological diagnosis. The accuracy of computed tomography (CT)-guided biopsy is significantly lower for small pulmonary nodules than for nodules > 1 cm^(^^5^^)^. Although minimally invasive video-assisted thoracoscopic surgery has come to be widely used for the diagnosis of small lesions, it is difficult for the surgeon to determine the exact location during surgery if the nodules are small and are located more than 2 cm from the pleural surface^(^^6^^)^. If they are deeper in the lung, palpation is necessary in order to locate them for excision^(^^7^^)^. For all other nodules that are potentially resectable through video-assisted thoracoscopic surgery, the preoperative location should be considered^(^^8^^)^.

There have been reports of various nodule localization techniques employing markers such as spiral hookwires, contrast media, cyanoacrylate, and methylene blue^(^^9^^)^. Localization using hookwires has been reported in larger studies, sometimes being performed in combination with methylene blue injection and sometimes being used in children^(^^10^^-^^12^^)^. Most of these marker systems have been customized for use in lung tissue or were not specifically designed for such use, having rather been designed for the localization of breast lesions.

Because of the difficulty in locating a pulmonary nodule intraoperatively and the increasing role that CT plays in identifying such nodules in clinical lung cancer screening, there has been extensive investigation to improve localization techniques in order to facilitate the resection of small nodules during video-assisted thoracoscopic surgery^(^^8^^)^.

**Figure 1:**
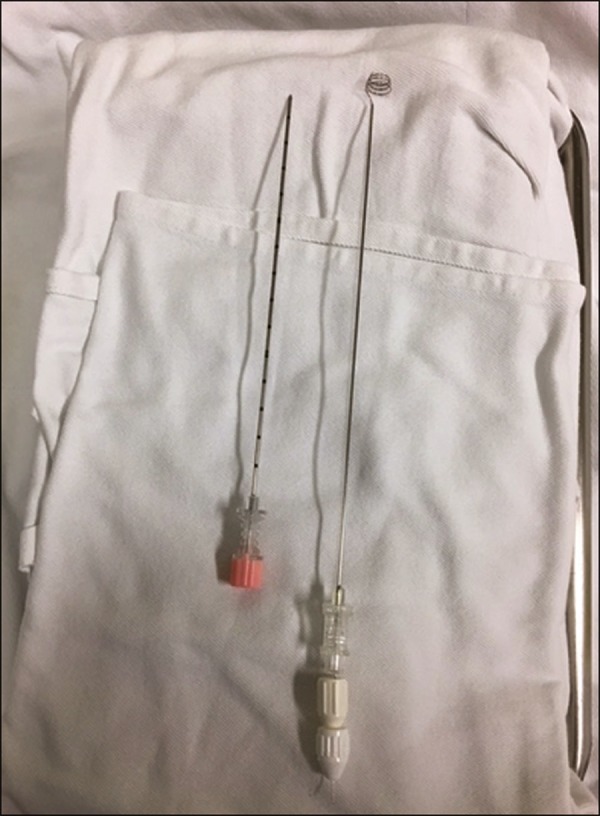
Nodule marker system composed of a coaxial needle and hookwire.

The marker system consists of an 18 G coaxial needle, an insertion device, and a nitinol hookwire. The hookwire can be repositioned if necessary, and the spiral tip provides firm anchoring in the lung tissue (Figure 1).

## PROCEDURE

**Figure 2:**
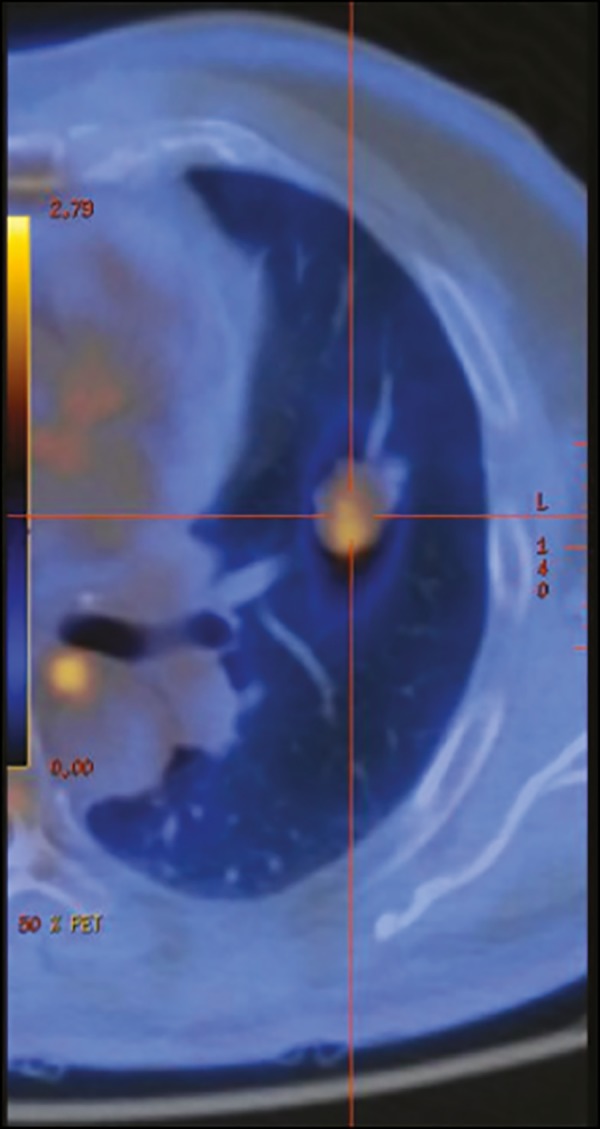
Axial PET/CT showing a pulmonary nodule with soft-tissue density and lobulated contours, located in the upper lobe of the left lung, with a SUVmax of 2.19.

Depending on the location of the nodule (Figure 2), patients are positioned in the CT scanner in such a way as to achieve a better assessment of the depth of the lesion, as well as to optimize the hookwire placement and angulation-in the supine, prone, or oblique position.

**Figure 3:**
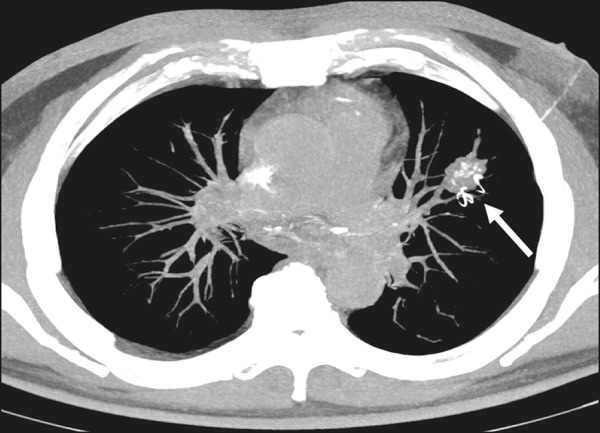
Axial MIP CT reconstruction showing the hookwire positioning, adjacent to the nodule with coarse calcifications (arrow).

After local infiltration with 2% lidocaine, the coaxial needle is inserted, under CT guidance, adjacent to or within the nodule, on the basis of an analysis made by a multidisciplinary team (comprising an interventional radiologist, a nuclear physician, and a thoracic surgeon). Upon withdrawal of the mandrel, the insertion device is used in order to implant the hookwire marker. The deployment requires no rotational movement. Before removing the coaxial needle and the insertion device, the correct position of the marker and the angle between the introducing needle and the pleural surface should be confirmed (Figure 3). Subsequently, a second CT scan is acquired to identify any immediate complications, such as pneumothorax and hematoma, or any other additional finding. Finally, the distance from the spiral tip to the intended target is evaluated.

**Figure 4:**
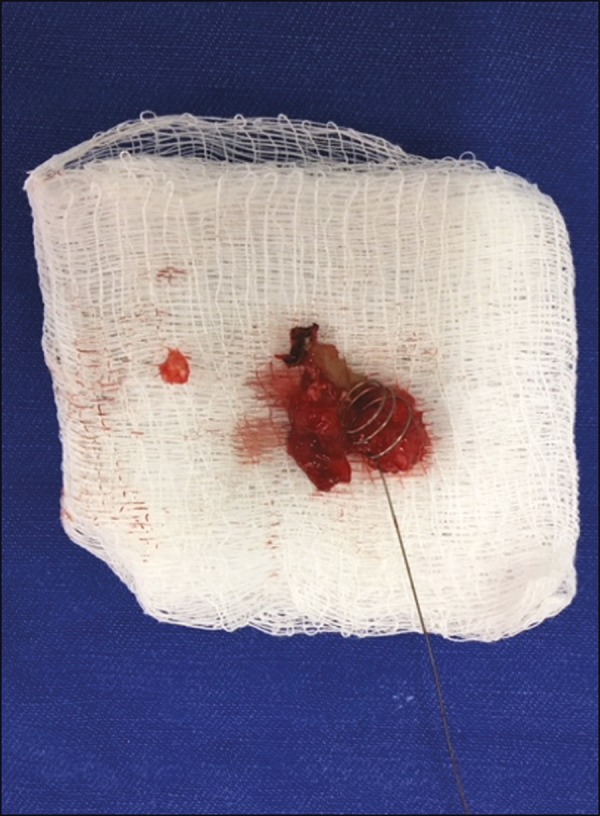
Macroscopic aspect of the surgical specimen (nodule) and hookwire marker.

After implantation of the hookwire, the patient is transported directly to the preoperative holding area. Any complaints are documented, and a brief report of the procedure, together with copies of the relevant CT images, is sent to the operating room. After video-assisted thoracoscopic surgery and excision of the nodule (Figure 4), the surgeon fills out a form documenting the position and accuracy of the guidewire location and a brief assessment of the system used.
